# Lack of Association between Stroke and Left Atrial Out-Pouching Structures: Results of a Case-Control Study

**DOI:** 10.1371/journal.pone.0076617

**Published:** 2013-10-07

**Authors:** Ji Young Ko, Young Dae Kim, Yoo Jin Hong, Hye-Jeong Lee, Jin Hur, Byoung Wook Choi, Ji Hoe Heo, Young Jin Kim

**Affiliations:** 1 Department of Radiology, Research Institute of Radiological Science, Severance Hospital, Yonsei University College of Medicine, Seoul, Korea; 2 Department of Neurology, Severance Hospital, Yonsei University College of Medicine, Seoul, Korea; 3 Division of Cardiovascular Radiology, Severance Cardiovascular Hospital, Yonsei University College of Medicine, Seoul, Korea; University of Münster, Germany

## Abstract

**Background and Purpose:**

Clinical significance of out-pouching structures of the left atrium (LA) as potential embolic sources remains unclear. We sought to evaluate the association between stroke and LA out-pouching structures.

**Methods:**

A case-control study was conducted to assess the prevalence of LA out-pouching structures in subjects with and without stroke. Case subjects were 270 stroke patients who had undergone cardiac CT. Control subjects were 270 age- and sex-matched patients without a history of stroke and who had undergone cardiac CT. Presence of LA out-pouching structures was determined by ECG-gated cardiac CT. The location of out-pouching structures was categorized as near Bachmann bundle, anterior, inferoseptal, inferior, and lateral. The prevalence, number and location of out-pouching structures and clinical characteristics were compared between the two groups.

**Results:**

One hundred sixty eight out-pouching structures were identified in 139 stroke patients (51%), while a total of 169 out-pouching structures were found in 155 control patients (57%) (p=0.1949). The prevalence of LA out-pouching structures with different locations was not significantly different between the stroke group and control group. In the stroke group, the prevalence of out-pouching structures was not significantly different by subtypes of ischemic stroke and the prevalence of LA out-pouching structures was not different between patients with atrial fibrillation (AF) and without AF.

**Conclusion:**

The left atrial out-pouching structures are commonly seen in a population with and without stroke with similar prevalence. Our study suggests that LA out-pouching structures are not significant risk factors of stroke.

## Introduction

Cardiogenic emboli have been considered to be the main causal factor in 20-40% of all stroke cases [1]. Stroke caused by intracardiac thrombi can be efficiently prevented by appropriate anticoagulation therapy. Therefore, the identification of a cardiac source of embolism in stroke patients is necessary to establish a treatment plan and to prevent recurrent stroke.

The ongoing development of cardiac computed tomography (CT) techniques has resulted in better visualization of cardiac anatomy and cardiac CT has become a potential noninvasive imaging modality in the detection of cardiac sources of embolism in stroke patients [2-4]. Recent studies using cardiac CT revealed that the prevalence of left atrial (LA) out-pouching structures including diverticula or accessory appendages is around 10-46% in the general population, a reason to consider these structures as an anatomic variant [5-9]. Several researchers have reported sporadic cases of out-pouching structures in the left atrium and have suggested that these structures might be a possible source of unexplained embolic stroke or atrial fibrillation (AF) [10-12]. However, until now, the exact relationship between LA out-pouching structures and stroke has not been studied on its own and still remains unclear [8,9].

In this study, we aimed to evaluate the prevalence, number, and location of LA out-pouching structures in patients with stroke in comparison to those of patients without stroke using ECG-gated cardiac CT and to reveal the relationship between these variant structures and stroke.

## Materials and Methods

### Ethics

This retrospective study was approved by our institutional review board. Informed consent was given by the patients for their information to be stored in the hospital database and used for research.

### Patient selection

We reviewed the records of consecutive patients who were included in the Yonsei stroke registry. Yonsei stroke registry is a prospective hospital-based registry for patients with acute ischemic stroke or transient ischemic attack [13]. The patients who registered in the stroke registry underwent brain CT or magnetic resonance (MR) imaging to exclude hemorrhages and other causes of symptoms and underwent at least one form of vascular imaging, such as conventional cerebral angiography, MR angiography, or CT angiography. Transesophageal echocardiography was part of the standard evaluation for cardiac or arterial embolic sources except in patients with decreased consciousness, poor systemic condition, tracheal intubation, or inability to accept an esophageal transducer. To evaluate possible coexistent coronary artery disease, cardiac CT was performed in patients who had more than two cardiovascular risk factors or who had cerebrovascular or peripheral arterial occlusive disease. Exclusion criteria of cardiac CT were 1) known coronary artery disease, 2) poor general condition, 3) impaired renal function, 4) failure to obtain informed consent.

From April 2009 to September 2011, 270 consecutive patients who had been admitted to our hospital with an episode of stroke and had undergone cardiac CT were included in our study and classified as the stoke group. The stroke group consisted of 179 men (mean age, 61.6 years; range, 20-85 years) and 91 women (mean age, 67.6 years; range, 46-92 years). For a control group, we selected 270 patients who had no history of stroke or transient ischemic attack from our cardiac CT database and who were age- and sex-matched with the stoke group. The indication for cardiac CT in the control group was clinically suspected coronary artery disease.

Clinical information including diabetes mellitus, hypertension, dyslipidemia, atrial fibrillation, and smoking status were obtained from electronic medical records. The subtypes of ischemic stroke in the stroke group were classified according to the Trial of Org 10172 in Acute Stroke Treatment criteria (TOAST criteria) [14]. The classification was as follows: 1) large-artery atherosclerosis, 2) cardioembolism, 3) small-artery occlusion, 4) stroke of other determined etiology, and 5) stroke of undetermined etiology. The final fifth category included patients with two or more possible causes of stroke identified, patients with negative evaluation, and patients with incomplete evaluation.

### Image acquisition protocol

CT scans were performed using a dual-source CT scanner (SOMATOM Definition Flash, Siemens, Forcheim, Germany). In the absence of contraindications, an oral β-blocker (50 mg of metoprolol tartrate; Betaloc, Yuhan, Seoul, Korea) was administered 1 hour prior to examination to reduce the heart rate in patients with heart rates above 65 bpm and a 0.3mg sublingual dose of nitroglycerin was administered just before the scan.

Scan delay times between the start of contrast agent injection and of scanning were determined by the timing bolus technique. After a bolus injection of 10 mL of iopamidol (Pamiray 370; 370mg iodine/mL; Dongkook Pharma, Seoul, Korea) followed by 20 mL of saline with 5 mL/s, optimal delay times were determined by automatic evaluation of the contrast enhancement in the ascending aorta. All CTs were performed with the triple-phase injection method (70 mL of iopamidol followed by 30 mL of 30% blended iopamidol with saline and 20 mL of saline at 5 mL/s).

The data acquisition mode was determined by heart rate (HR). In cases of a regular HR < 60 bpm, scans were performed with the prospectively electrocardiogram (ECG)-triggered high-pitch spiral mode. In cases of a regular HR between 60 and 75 bpm, scans were performed with the prospectively ECG-triggered axial mode targeting mid-diastole, and in cases of a regular HR ≥ 75 bpm, with the prospectively ECG-triggered axial mode targeting end-systole [15]. If frequent ectopic beats or irregular HR were seen during prescan ECG monitoring, scans were performed with retrospectively ECG-gated data acquisition with ECG-based tube current modulation. The scan parameters were as follows: a gantry rotation time of 0.28 s, a tube voltage of 80, 100, or 120 kV and a tube current-time product of 200 to 450 mAs decided by the patient’s body mass index, and a collimation width of 0.6 mm.

Images were reconstructed with a slice thickness of 0.75 mm and an increment of 0.5 mm using a medium kernel of B36f and sent to a workstation (Aquaris iNtuition, TeraRecon Inc. San Mateo, CA). Images were evaluated with axial reconstruction images, volume-rendering images, and interactive multiplanar reformat images.

### Image analysis

Two experienced radiologists (J.Y.K. and Y.J.K., with 3 years and 9 years of experience with cardiac CT) reviewed the images via consensus reading. Each reader was blinded to the patient’s group and other clinical information. Evaluation of the LA out-pouching structures was based on the axial and short axis CT images. In general, an accessory LA appendage was defined if the structure had a discernible ostium and irregular contours suggestive of the presence of pectinate muscles, and LA diverticulum was defined if the structure had a saclike shape with a broad-based ostium and a smooth contour at the body portion. However, the differentiation of an accessory LA appendage from LA diverticulum can be difficult because the defining features may be subtle and some cases may express features of both appendages and diverticula [5,16]. Thus we recorded these structures together as out-pouching structures.

**Figure 1:**
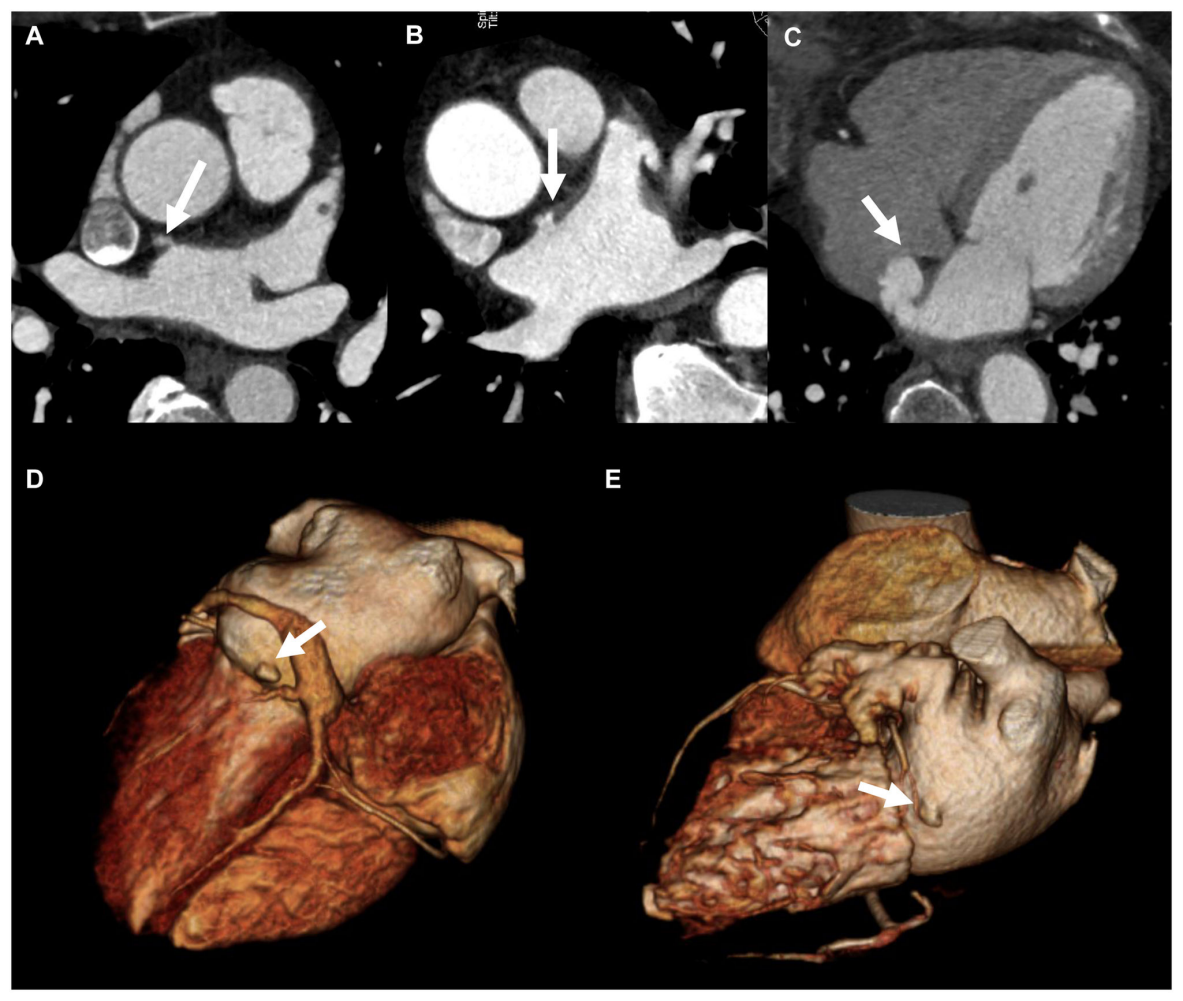
Location of LA out-pouching structures was divided into five categories: (A) near Bachmann bundle, (B) anterior, (C) inferoseptal, (D) inferior, and (E) lateral.

The presence, number and location of LA out-pouching structures were recorded in all subjects, regardless of them being either an accessory LA appendage or LA diverticulum. The location of the out-pouching structures was divided into five categories: 1) near Bachmann Bundle, 2) anterior (excluding 1), 3) inferoseptal (septal location, not included in 1), 4) inferior (inferior wall of the left atrium), 5) lateral (located in the LA lateral wall above the mitral valve) (Figure 1).

### Statistical analysis

Under the assumption that the prevalence of LA out-pouching structures was 40% in the control group and 55% in the stroke group, the sample size was calculated as 462 to have a 90% power and to detect a difference between arms with a two-sided type I error rate of 5%. The calculated power in an unmatched sample size of 270 in each group with a 1:1 comparison (total sample size of 540) was 94%.

The prevalence and location of out-pouching structures were compared between the stroke group and control group. In addition, we investigated whether the prevalence of out-pouching structures were statistically different according to the presence of atrial fibrillation and stroke subtypes based on the TOAST criteria. These categorical prevalence data are presented as percentages, and comparisons were performed with the chi-square test.

All statistical analyses were performed using MedCalc for Windows, version 12.3.0.0 (MedCalc Software, Mariakerke, Belgium).

## Results

### Patient Characteristics

The clinical characteristics of the stroke group and control group are summarized in Table 1.

**Table 1:** Baseline characteristics of case and control subjects.

	Stroke group (n=270)	Control group (n=270)	*P*-value
Age, mean ± SD, y	63.6±11.2	63.6±11.2	
Male	179 (66.3)	179 (66.3)	
Hypertension	202 (74.8)	131 (48.5)	<0.00001
Diabetes Mellitus	83 (30.7)	59 (21.9)	0.02456
Dyslipidemia	77 (28.5)	64 (23.7)	0.2404
Smoking history	106 (39.3)	9 (3.3)	<0.00001
Atrial fibrillation	34 (12.6)	35 (13.0)	>0.9999

Note. Data in parentheses are percentages.

The prevalence of hypertension, diabetes mellitus, and smoking status were significantly higher in the stroke group than in the control group. However, atrial fibrillation was observed with similar prevalence in both groups.

### Prevalence of LA out-pouching structures

A total of 168 out-pouching structures were found in 139 out of 270 (51%) patients in the stroke group, whereas 169 out-pouching structures were found in 155 out of 270 patients (57%) in the control group. There was no statistically significant difference in the prevalence of out-pouching structures (p=0.1949). No thrombi were found within these structures. Table 2 summarizes the locations of the LA out-pouching structures. The most common location in the stroke group was the near Bachmann Bundle location, while the control group at the lateral wall. However, there were no significant differences in terms of out-pouching structure locations between the two groups.

**Table 2:** Prevalence and location of LA out-pouching structures.

	Stroke group (n=270)	Control group (n=270)	*P*-value
Prevalence of out-pouching structures (%)	139/270 (51)	155/270 (57)	0.1949
Near Bachmann bundle	65/270 (24)	51/270 (19)	0.1731
Anterior	36/270 (13)	52/270 (19)	0.0825
Inferoseptal	14/270 (5)	9/270 (3)	0.3940
Inferior	7/270 (3)	3/270 (1)	0.3383
Lateral	46/270 (17)	54/270 (20)	0.4381
Patients with multiple LA out-pouching structures	25/270 (9)	31/270 (11)	0.4803

Note. Data in parentheses are percentages.

Thirty-four of 270 stroke patients had atrial fibrillation. The prevalence of out-pouching structures was not significantly different between patients with and without atrial fibrillation (p=0.9989) (Table 3). In the stroke group, the presence of out-pouching structures did not differ according to the stroke mechanism based on the TOAST classification (Table 4).

**Table 3:** Prevalence of atrial fibrillation (AF) and LA out-pouching structures in the stroke group.

	Patients with AF (n=34)	Patients without AF (n= 236)
Patients with LA out-pouching structures (n=139)	17 (50)	122 (52)
Patients without LA out-pouching structures (n=131)	17 (50)	114 (48)

Note. Data in parentheses are percentages.

*p-value = 0.9989

**Table 4:** Prevalence of LA out-pouching structures according to the TOAST classification in 270 stroke patients.

Subtypes	Total (n=270)	Patients with LA out-pouching structures (n=139)	Patients without LA out-pouching structures (n=131)
Large artery atherosclerosis	67	33 (49)	34 (51)
Cardioembolism	68	38 (56)	30 (44)
Small artery occlusion	48	25 (52)	23 (48)
Other determined etiology	5	3 (60)	2 (40)
Undetermined etiology	82	40 (49)	42 (51)

Note. Data in parentheses are percentages.

*p-value = 0.9018

## Discussion

To our knowledge, this is the first study which assesses the prevalence and characteristics of LA out-pouching structures using ECG-gated cardiac CT in a cohort with stroke and compares them with a control group of patients without stroke. This study showed no difference in the prevalence or location of LA out-pouching structures in patients with stroke compared to control patients without stroke. Also, the prevalence of LA out-pouching structures had no relationship with subtypes of ischemic stroke. In addition, this study showed that LA out-pouching structures was not significantly associated with AF.

In this study, the prevalence of out-pouching structures was high for both groups (stroke group, 51%; control group, 57%). The prevalence of LA out-pouching structures was slightly higher than previously reported prevalence values of accessory LA appendages and LA diverticula that were around 10%-46% [5-7,9,17]. The location of LA out-pouching structures was concordant with previous results where they were commonly seen in the right anterosuperior wall and left lateral wall of the LA although somewhat different classifications have been used to categorize the location of LA out-pouching structures in previous studies [5,8,9,16].

There is still debate about the clinical significance of LA out-pouching structures as a potential cause of cardioembolic stroke. Previous reports regarding accessory LA appendages or diverticula suggested that these out-pouching structures might be associated with an increased risk of thromboembolic disease because of low flow and turbulence within the sac-like structure [5,16]. There have been a few case reports about the aggressive treatment of these out-pouching structures that were regarded as a potential cardioembolic source [10,18,19]. We hypothesized that if the LA out-pouching structures were a possible embolic source, the prevalence of these structures would be higher in patients who were classified as stroke with undetermined etiology than in others that were differently classified. However, the present study revealed that there was no evidence to suggest that these out-pouching structures might be a substrate for stroke causing thrombogenesis. According to the cardiac CT study done by Killeen et al., accessory LA appendages have significant contractile properties and thus, the likelihood of thrombogenesis due to low flow or turbulence may be less than expected [17]. Together with our results, LA out-pouching structures may be bystanders, rather than offenders, in the development of stroke.

In our study, when patients with stroke were divided into patients with AF and without AF, there was no significant difference in the presence of LA out-pouching structures. Several recent studies evaluated the prevalence of LA out-pouching structures between patients with AF and those with sinus rhythm [8,9,20]. These studies found no significant differences in the prevalence of LA out-pouching structures between the AF group and control group. Our results are concordant with results from former studies about the prevalence of LA out-pouching structures in patients with or without AF [8,9].

The limitations of this study should be mentioned. First, we did not evaluate the accessory LA appendages and diverticula separately but evaluated together as “LA out-pouching structures” because the differentiation between the two structures is difficult to do with CT appearance. An example is the presence of pectinate muscles in structures of small size. Therefore, the possible difference in clinical significance between the accessory LA appendage and diverticulum could not be evaluated in this study. Second, we could not reveal how the size of LA out-pouching structures affected the patients because most of the LA out-pouching structures detected in our study population were very small (less than 1cm) and the largest one measured just 1.2cm in diameter. In previous reports, the thrombogenic or arrhythmogenic potential of LA out-pouching structures were initially suggested in patient cases with large accessory LA appendages or diverticula [10,19,21]. Therefore, the effect of LA out-pouching structure size should be further investigated. Third, the relationship between LA size or volume and LA out-pouching structures could not be evaluated in this study because CT data were acquired at different time points of the cardiac cycle (end-systole or mid-diastole) in each patient according a patient’s heart rate. Fourth, the sample size in this study was determined by only considering the power needed to detect a difference in prevalence of the entire LA out-pouching structures between the stroke and control groups. Therefore, it might be underpowered to detect differences between location and stroke subtypes. Lastly, this study may have some sampling biases in its study design. For the stroke group, patients who have a higher risk of non-cardioembolic stroke may be selected. For the control group, there is a possibility that patients with unrecognized brain infarction that was clinically silent were included in the study.

In conclusion, LA out-pouching structures are commonly seen in the population with and without stroke with similar prevalence. We found no association between the prevalence and location of LA out-pouching structures and stroke in this case-control study. 
